# Structure and applications of novel influenza HA tri-stalk protein for evaluation of HA stem-specific immunity

**DOI:** 10.1371/journal.pone.0204776

**Published:** 2018-09-27

**Authors:** I-Na Lu, Anna Kirsteina, Sophie Farinelle, Stéphanie Willieme, Kaspars Tars, Claude P. Muller, Andris Kazaks

**Affiliations:** 1 Department of Infection and Immunity, Luxembourg Institute of Health, Esch-sur-Alzette, Luxembourg; 2 Latvian Biomedical Research and Study Centre, Riga, Latvia; 3 Department of Molecular Biology, Faculty of Biology, Riga, Latvia; 4 Laboratoire National de Santé, Dudelange, Luxembourg; Icahn School of Medicine at Mount Sinai, UNITED STATES

## Abstract

Long alpha helix (LAH) from influenza virus hemagglutinin (HA) stem or stalk domain is one of the most conserved influenza virus antigens. Expression of N-terminally extended LAH in *E*. *coli* leads to assembly of α-h elical homotrimer which is structurally nearly identical to the corresponding region of post-fusion form of native HA. This novel tri-stalk protein was able to differentiate between group 1 and 2 influenza in ELISA with virus-infected mice sera. It was also successfully applied for enzyme-linked immunospot assay to estimate the number of HA stem-reactive antibody (Ab)-secreting cells in mice. An in-house indirect ELISA was developed using a HA tri-stalk protein as a coating antigen for evaluation of HA stem-specific Ab levels in human sera collected in Luxembourg from 211 persons with occupational exposure to swine before the pandemic H1N1/09 virus had spread to Western Europe. Our results show that 70% of these pre-pandemic sera are positive for HA stem-specific Abs. In addition, levels of HA stem-specific Abs have positive correlation with the corresponding IgG titers and neutralizing activities against pandemic H1N1/09 virus.

## Introduction

With the annual epidemics causing 3 to 5 million cases of severe illness and up to 650 000 deaths per year human influenza virus remains a significant health and economic burden worldwide (WHO 2018: http://www.who.int/en/news-room/fact-sheets/detail/influenza-(seasonal)) [1]. Apart from the seasonal epidemics which are caused by antigenic drift of influenza viruses, the introduction of novel virus variants from the zoonotic pool via antigenic shift can result in viruses capable of initiating human pandemics [2]. In the past hundred years, four influenza pandemics have spread in the human population [3], the deadliest of them being the 1918 influenza pandemic when the mortality reached up to 50 million cases [4]. Some avian influenza strains, such as H5N1, H7N9 and H6N1, represent a risk that if they become transmissible among humans new pandemic influenza strains will emerge inducing even more devastating effects to the public health [5–7]. Rapid diagnostics can speed up the treatment decreasing the spreading of the influenza virus and is one of the key components of pandemic preparedness.

Influenza virus hemagglutinin (HA) is the major surface antigen of the virion and the primary target of virus neutralizing antibodies (Abs) [8]. HA is a homotrimeric surface glycoprotein, with each monomer consisting of two disulfide-linked subunits (HA1, HA2), resulting from the proteolytic cleavage products of a single HA precursor protein. The HA1 chain forms a membrane-distal globular head and a part of the membrane-proximal stem region. The HA2 chain represents the major component of the stem region [9]. The head of HA mediates receptor binding while the membrane-anchored stem is the main part of membrane fusion machinery [10]. Neutralizing Ab responses are mainly targeted to the immunodominant head domain of HA [11]. However, because of the high genetic plasticity of the head region epitopes [12] Ab responses are strain-specific and lack broad cross-reactivity with different HA subtypes [11]. In contrast, sequence and structure of the subdominant HA stem are much more conserved across different influenza subtypes and broadly neutralizing Abs against this domain are considered promising therapeutic tools against various influenza virus strains [8], [13]. Indeed, there are some Abs known that cross-react with HA stem from all influenza A subtypes [14] or even with HA stem from both influenza A and B viruses [15].

One of the most conservative HA stem regions is a 55 amino acid (aa) long alpha helix (LAH) which is currently under intensive investigation as a potential universal influenza virus antigen [16–17]. Recently, we demonstrated that the LAH, as well as its N-terminally extended variant (72 aa), incorporated into hepatitis B virus core (HBc) particles is highly immunogenic in mice [18–19]. Expression of the extended LAH antigen in *Escherichia coli* resulted in efficient synthesis of a soluble product. We purified and crystallized the protein and determined its three-dimensional structure, revealing formation of an α-helical trimer (referred to as tri-stalk protein) highly similar to the corresponding region of native HA in its post-fusion form.

We have developed an in-house indirect ELISA using the HA tri-stalk protein as immobilized antigen to evaluate HA stem-specific Ab levels in mice and human sera. In addition, tri-stalk protein was successfully used in an enzyme-linked immunospot (ELISPOT) assay to estimate numbers of HA stem-reactive Ab-secreting cells in mice. To extend the previous study [20] and examine the level of pre-existing Abs in this population, our in-house ELISA was used to test human sera pool collected from people with direct occupational contacts to swine before the pandemic H1N1/09 virus had spread to Western Europe. Our results show that 70% of these pre-pandemic sera are positive for group 1 HA stem-specific Abs. In addition, the levels of HA stem-specific Abs have positive correlation with the corresponding IgG titers and neutralizing activities against pandemic H1N1/09 virus. Taken together, we have demonstrated that the novel HA tri-stalk protein is a useful tool for evaluation of HA stem-specific immunity in both mice and humans.

## Materials and methods

### HA tri-stalk protein

The tri-stalk protein encoding HA amino acids 403–474 of the pandemic strain A/Luxembourg/43/2009(H1N1) was PCR-amplified and cloned in the pETDuet-1 vector (*Novagen*) using *Nco*I and *Bsp*TI restriction sites. The construct was expressed in *Escherichia coli* BL21 (DE3) cells using a standard protocol (https://assets.thermofisher.com/TFS-Assets/LSG/manuals/oneshotbl21_man.pdf). Briefly, cells were cultivated in 2×TY medium in Erlenmeyer flasks under continuous shaking (200 rpm) at +37°C. When optical density OD_A540_ = 0.6–0.8 was reached IPTG was added to a final concentration of 0.1 mM. After another 3 h, the cells were collected by low-speed centrifugation and stored at -20°C until use.

For purification of the antigen, the cells were disrupted in lysis buffer A containing 20 mM Tris HCl, pH 8.0, and 100 mM NaCl (6 mL of buffer per 1 g of cells) by sonication. The soluble fraction was isolated by centrifugation (18,000 × *g*, 30 min). The supernatant was subjected to thermal treatment for 30 min at 55°C and centrifuged again. The soluble fraction was applied to a weak anion exchange HiPrep 16/10 DEAE FF column in buffer A. Bound protein was linearly eluted (4 mL/min) with buffer B (20 mM Tris HCl, pH 8.0, 1 M NaCl) in 3 column volumes (CVs) until concentration of 0.6 M NaCl was reached. Selected fractions were pooled, diluted twice with 20 mM Tris HCl, pH 8.0, and applied onto a strong anion exchange column MonoQ 5/50 GL in buffer A (1 mL/min). Column-bound proteins were linearly eluted with buffer B in 10 CVs until concentration of 0.5 M NaCl was reached. Finally, protein was subjected to size exclusion chromatography on a Superdex 200 10/300 GL column in 20 mM Tris HCl, pH 8.0, 200 mM NaCl at 0.5 mL/min. All runs were performed at room temperature and monitored using the ÄKTA FPLC chromatography system (Amersham Biosciences). Purified HA tri-stalk protein was aliquoted and stored at -20°C until use.

### Crystallization and structure determination

Prior to crystallization, purified HA tri-stalk protein was concentrated to 10 mg/mL in 20 mM Tris HCl, pH 8.0, 200 mM NaCl, using Amicon 10 kDa MWCO filter (Millipore), and crystallized by mixing with equal volume of precipitant solution (1 μL each) using the sitting-drop vapor-diffusion technique. The HA tri-stalk crystals used for data collection were obtained at 2.0 M ammonium sulfate and 0.1 M Bis-Tris, pH 5.5. For data collection, the crystals were flash-frozen in liquid nitrogen, after brief soaking in a cryoprotectant containing reservoir liquor and 30% glycerol. Datasets were collected at MAX-lab beamline I911-3 (Lund University, Sweden).

The diffraction images were integrated with MOSFLM [21], and scaled using SCALA [22] from the CCP4i program suite [23]. The crystal structure of HA tri-stalk protein was solved by molecular replacement with PHASER [24], using the corresponding fragment of structure of influenza HA at the pH of membrane fusion as the search model [25]. Initially the model was generated in BUCCANEER [26]. The structure was manually adjusted and validated using the COOT software program [27] and refinement was carried out in REFMAC [28]. The HA tri-stalk 3D structure was visualized in PyMOL (The PyMol Molecular Graphics System, Version 1.8 Schrödinger, LLC). SSM-superimposition was carried out in COOT. Statistics for data collection and structure refinement are presented in Table 1. Coordinates and structure factors are deposited in the Protein Data Bank with accession code 6GOL.

**Table 1:** Data collection and refinement statistics.

**Data collection and scaling**	
Beamline	I911-3
Wavelength (Å)	1.00000
Space group	P6_3_
Unit cell parameters (Å)	a = b = 41.80, c = 90.12
Resolution (Å)	23.12–1.7 (1.79–1.7)
Observations	36458
Unique reflections	9803 (1439)
Completeness (%)	99.7 (100)
I/σ_I_	10 (1.8)
R_merge_	0.063 (0.664)
Multiplicity	3.7 (3.7)
**Refinement statistics**	
R_work_	0.17771 (0.322)
R_free_	0.21914 (0.320)
Average B-value (Å^2^)	36.711
Wilson B-value (Å^2^)	22.7
Protein atoms	538
Waters	57
**RMSD from ideal geometry**	
Bond length (Å)	0.024
Bond angles (°)	2.009
**Ramachandran outliers**	
Residues in favored regions (%)	100

### Mouse infection and immunization

Female BALB/c mice were purchased from Harlan Laboratories, Inc., the Netherlands. All mice experiments were performed in accordance with protocols approved by the Animal Welfare Structure of Luxembourg Institute of Health and by the Minister of Agriculture, Viticulture and the Consumer Protection of the Grand Duchy of Luxembourg (Ref. LNSI-2014-02). All experiments with live influenza virus were performed in a biosafety level 3 (BSL-3) containment facility, and mice were kept in positive-pressured isocage system.

For virus infection, 10-week-old BALB/c mice (n = 8 mice/group) were infected intranasally with a sublethal dose (8×10^4^ half maximal tissue culture infectious dose; TCID50) of pandemic H1N1 influenza strain A/Luxembourg/46/2009 (pH1N1), A/Texas/36/91 seasonal H1N1 (sH1N1), A/Puerto Rico/8/34 H1N1 (PR8), A/Lux/01/2005 seasonal H3N2 (sH3N2), A/Aïshi/68 H3N2 (X-31), or B/Lee/40 virus in 50 μL phosphate-buffered saline (PBS), after anesthesia with isoflurane. We evaluated and weighed the mice every day at 10:00 AM for 14 days following sub-lethal infection, and no adverse or unexpected events were observed.

For immunization of mice, LAH domain-encoding sequence from pH1N1/09 strain was genetically fused to the N-terminus of bacteriophage PP7 coat protein gene separated by a short GSG-encoding linker, designated as LAH-PP7. This fusion gene was cloned and expressed as described for HA tri-stalk construct. The cells were disrupted in lysis buffer A by sonication and centrifuged at 18,000 × *g* for 30 min. Insoluble fraction was washed twice with lysis buffer and sonication repeated with the addition of 0.25 M urea in buffer A. After centrifugation, the soluble fraction containing LAH-PP7 fusion protein was loaded onto a Superdex 200 10/300 GL column in buffer A and target protein fractions were collected.

8-week-old female BALB/c mice (n = 8 mice/group) were vaccinated three times with 2-week interval intraperitoneally with 200 μL of 30 μg LAH-PP7 fusion protein, tri-stalk protein or PBS in aluminium hydroxide gel adjuvant (Brenntag Biosector, Denmark). One week after the final boost, mice were killed by intraperitoneal injection of 200 mg/kg of sodium pentobarbital. Blood and spleens were collected for HA tri-stalk protein based ELISA and ELISPOT assay, respectively.

### Enzyme linked immunosorbent assay (ELISA)

The indirect ELISA conditions were optimized by checkerboard titration using serial dilutions of HA tri-stalk protein tested against serial dilutions of positive and negative sera. Briefly, the HA tri-stalk protein was coated overnight at 4°C to 384-well microtiter plates (Greiner Bio-One GmbH, Austria) using concentrations of 0.156, 0.313, 0.625, 1.25, 2.5, 5.0 and 10.0 μg/mL. After washing, free binding sites were saturated at room temperature with 1% BSA in Tris buffer. After 2 h, plates were washed again and diluted mouse (starting with 100-fold dilutions) or human sera (starting with 300-fold dilutions) were added and incubated for 90 min at room temperature. Binding was assessed by alkaline phosphatase-conjugated goat anti-human IgG (1/1000 dilution, Southern Biotech, Birmingham, AL) and 4-nitrophenyl phosphate disodium salt hexahydrate (Phosphatase substrate, Sigma-Aldrich, Germany). Absorbance was measured at 405 nm (SpectraMax Plus 384 Microplate reader), after 60 min of incubation at 37°C. Sera from naïve mice and low-titer sera from young children served as negative controls.

3.5 μg/mL purified recombinant HA protein from A/Texas/36/1991 (HA H1/91) and A/California/4/2009 (HA H1/09, both purchased from Sino Biological Inc.) were coated respectively to the plates following the above ELISA protocol. Positive and negative control sera were used on all plates for normalization between plates.

Influenza-specific IgG response in mice immunized with LAH-PP7 was measured by indirect ELISA using the following purified recombinant group 1 and group 2 HA antigens: A/California/4/2009 (Cal09; H1), A/Japan/305/57 (JP57; H2), A/Perth/16/2009 (Perth09; H3), A/Swine/Ontario/01911-1/99 (Onta99; H4), A/Vietnam/1203/04 (VN04; H5), A/Netherlands/219/2003 (Neth03; H7), A/Hong Kong/1073/99 (HK99; H9), A/Jiangxi-Donghu/346/2013 (JX13; H10), A/mallard /Alberta/294/1977 (Alb77; H11), A/mallard/Astrakhan/263/1982 (Astrak82; H14), A/duck/AUS/341/1983 (AUS83; H15) and A/black-headed gull/Sweden/5/99 (SW99; H16), all purchased from Sino Biological Inc.

### Enzyme-linked immunospot (ELISPOT)

The numbers of Ab-secreting cells under stimulation by HA tri-stalk protein were counted using commercial mouse IgG1 and IgG2a single-color ELISPOT assay kits (Cellular Technology Limited, Cleveland, OH) as per the manufacturer’s instructions. Freshly isolated splenocytes from mock or LAH-PP7 immunized mice were pre-stimulated for 4 days at 37°C in a 7% CO_2_ incubator, the cells were then transferred to capture Ab-coated polyvinylidene fluoride (PVDF) 96-well plates (5×10^5^ cells/well). Following incubation for 20–24 h at 37°C in a 5% CO_2_ incubator, the cell suspensions were removed. All wells were washed twice with PBS and twice with 0.05% Tween-PBS, anti-murine IgG1 or IgG2a detection solution was added, and the plates were incubated for 2 h at room temperature. After 3 washes with 0.05% Tween-PBS, tertiary solution (Streptavidin-alkaline phosphatase conjugate) was added at 80 μL per well for 1 h at room temperature. Following four more washes, 80 μL of blue developer solution (Substrate mix) was added for 15–20 min at room temperature in the dark to yield colored spots. Finally, the reaction was stopped by thoroughly rinsing with tap water. The plates were air-dried and stored in the dark until analysis. The number of spots was counted using the ImageQuant software (Molecular Dynamics, Sunnyvale, CA). The average number of spot-forming cells was adjusted to 1×10^6^ splenocytes.

### Donors and serum samples

Swine workers (SW) are defined in this study as persons whose professions involve direct contact with swine. 211 SW, including pig farmers, slaughterhouse workers and veterinarians, aged 18 to 94 years, were recruited to donate serum samples prior to the outbreak of pandemic H1N1/09 as described before [20]. Pre-pandemic sera from 71 non-swine workers (non-SW) were also collected as control. Informed consent was gathered from all participants enrolled. The study was approved by the National Ethical Committee for Research in Humans of the Grand Duchy of Luxembourg.

### Microneutralization assay

Heat inactivated (30 min at 56°C) and receptor destroying enzyme (RDE, purchased from DENKA SEIKEN UK Ltd, Coventry, UK) treated sera were tested by microneutralization assay against influenza A/Luxembourg/43/2009 (H1N1/09) virus according to the World Health Organization (WHO) protocol (Serological Diagnosis of Influenza by Microneutralization Assay. WHO 2010). Equal volumes of serum and virus diluted to 2×10^3^ median tissue culture infective doses (TCID_50_) per milliliter were incubated for 1 h at 37°C. The mixture was added to a confluent layer of Madin-Darby canine kidney (MDCK) cells in 96-well tissue culture plates (1.5×10^4^ cells/well) and incubated for 20 h. After fixation with ice-cold 80% acetone in PBS for 15 min, anti-influenza A NP mouse monoclonal Ab (1:1000 dilution) was added to the monolayers for 1 h. Virus‐infected cells were detected using an alkaline phosphatase conjugated with anti-mouse second step Ab. The optical density value of half-maximal effective dose (EC50) was then determined by the following equation: [(average OD_405_ of virus control wells) − (average OD_405_ of cell control wells)]/2. The reciprocal serum dilution corresponding to EC50 is the 50% neutralizing Ab titer for the serum sample.

### Statistical analysis

GraphPad Prism software was used for statistical evaluation of data. One-way ANOVA followed by Holm's test was applied for statistical analysis (*p<0.01, ***p<0.0001, ****p<0.00001). Spearman correlation test was employed to determine correlations.

## Results

### Purification of HA tri-stalk protein

**Fig 1:**
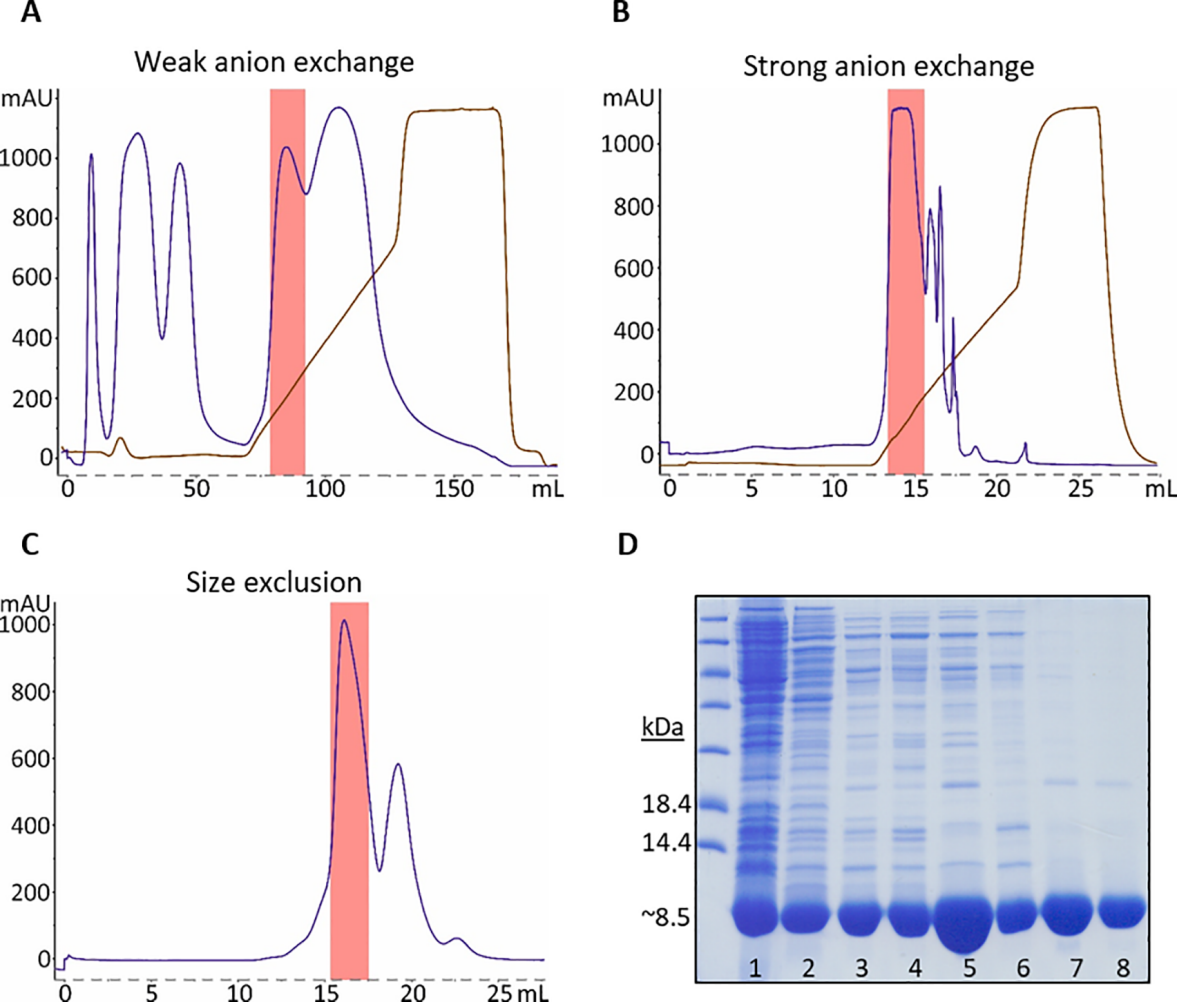
Purification of HA tri-stalk protein. Chromatography profiles of protein fractionation on DEAE FF (A), MonoQ (B) and Superdex (C) columns. Blue lines represent protein peaks while brown lines correspond to salt gradient. HA tri-stalk containing fractions are marked by red squares. (D) Coomassie-stained PAAG illustrating individual purification steps: 1 and 2, supernatant before and after thermal treatment, respectively; 3–4, DEAE column fractions; 5–6, MonoQ column fractions; 7–8, Superdex column fractions.

Our long-term research goal is exposition of the LAH region on virus-like particles (VLPs) in its native trimeric conformation. To assess structural and immunological properties of different LAH derivatives a number of constructs containing the LAH region have been compared for expression and solubility in *E*. *coli*. These peptides had the same C-terminus but differed by their N-terminal extensions. The stem peptide encoding HA amino acids 403–474 was the longest fragment (Mw = 8.5 kDa) which was efficiently produced in a soluble form and was selected for downstream applications as HA tri-stalk protein. For purification, we developed a fast and efficient method involving a combination of three chromatography steps: a weak anion-exchange, a strong anion-exchange and size-exclusion columns (Fig 1A–1C). Standard expression conditions generated high-levels of soluble tri-stalk protein (Fig 1D, lane 1). To simplify the purification procedure, the soluble protein mixture was subjected to thermal treatment at 55°C. As expected, a lot of contaminating proteins precipitated under these conditions while the majority of target protein remained in solution (Fig 1D, lane 2). Next, the protein solution was loaded on a weak anion exchange DEAE column. While the majority of contaminants passed through the column the tri-stalk protein was efficiently bound and eluted with ~200 mM salt (Fig 1A and 1D, lanes 3–4). The solution was then loaded on a strong anion exchanger MonoQ which further improved the purity of the tri-stalk protein (Fig 1B and 1D, lanes 5–6). Finally, protein was subjected to size-exclusion chromatography where two major peaks were observed (Fig 1C). Both peaks contained predominantly target protein, but only the first peak corresponded to the trimer size (Mw = 25.6 kDa) according to its elution volume, while the second peak seemed to contain non-assembled product and was discarded. The final product had at least 90% purity (Fig 1D, lanes 7–8) and was further used for structural and diagnostic studies.

### HA tri-stalk protein structure

The crystal structure of HA tri-stalk protein was solved to 1.7 Å resolution using molecular replacement technique. The space group was P6_3_ and the HA tri-stalk protein was found to be located on the crystallographic 3-fold axis. Therefore, the crystallographic asymmetric unit contained one protein molecule. The final model included a single protein chain with 65 residues and 57 water molecules. The first 3 N-terminal and 3 C-terminal residues were not included in the final model due to poor electron density.

**Fig 2:**
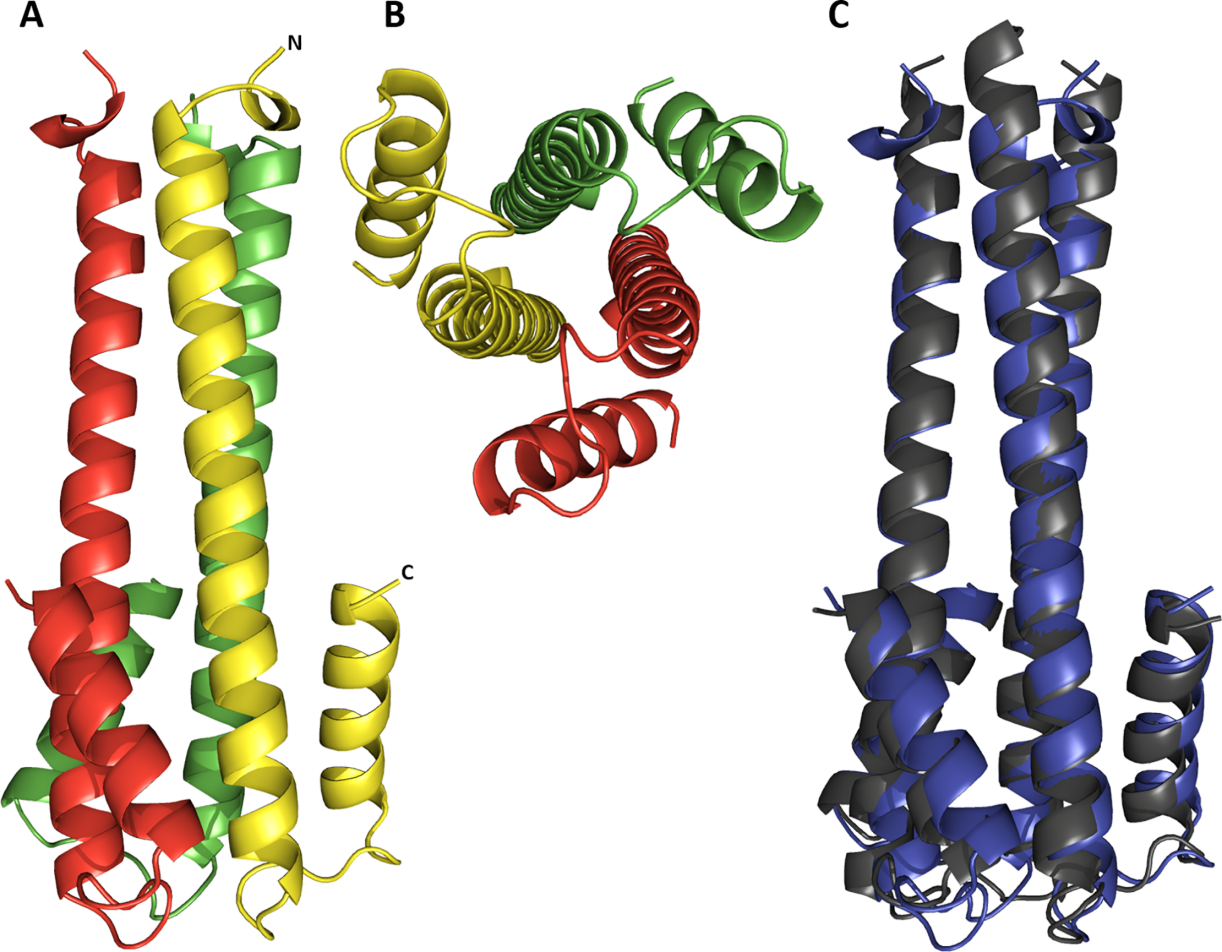
The structure of HA tri-stalk at pH 5.5. Side (A) and top (B) view on tri-stalk protein. Individual monomers are colored in red, green and yellow. (C) Structural alignment of HA tri-stalk protein (blue) with the corresponding stem fragment of native post-fusion HA (grey) (PDB code 1HTM, residues 63 to 127). Only matching sequence length is shown for clarity.

The structure revealed that HA tri-stalk is an elongated trimer with the length of approximately 63 Å (Fig 2A and 2B). Each monomer consists of a long α-helix at the N-terminus, shorter antiparallel α-helix at the C-terminus, and a small flexible loop region on the surface of the protein connecting both helices. The end of the N-terminus extends away from the rest of the protein. The central long α-helices form a tripartite coiled coil with the short α-helices packing along them and forming a 6-helix bundle-like structure. The protein clearly matched the structure of the corresponding stem fragment of the native HA at its low pH-induced post-fusion conformation [25]. However, it displayed some minor deviations from the wild-type HA in the loop region and at the termini of the helices with Cα atom root-mean-square deviations (RMSDs) of about 0.94 Å (60 residues of one monomer were aligned) (Fig 2C).

### HA tri-stalk reactive Abs in influenza virus infected mice

**Fig 3:**
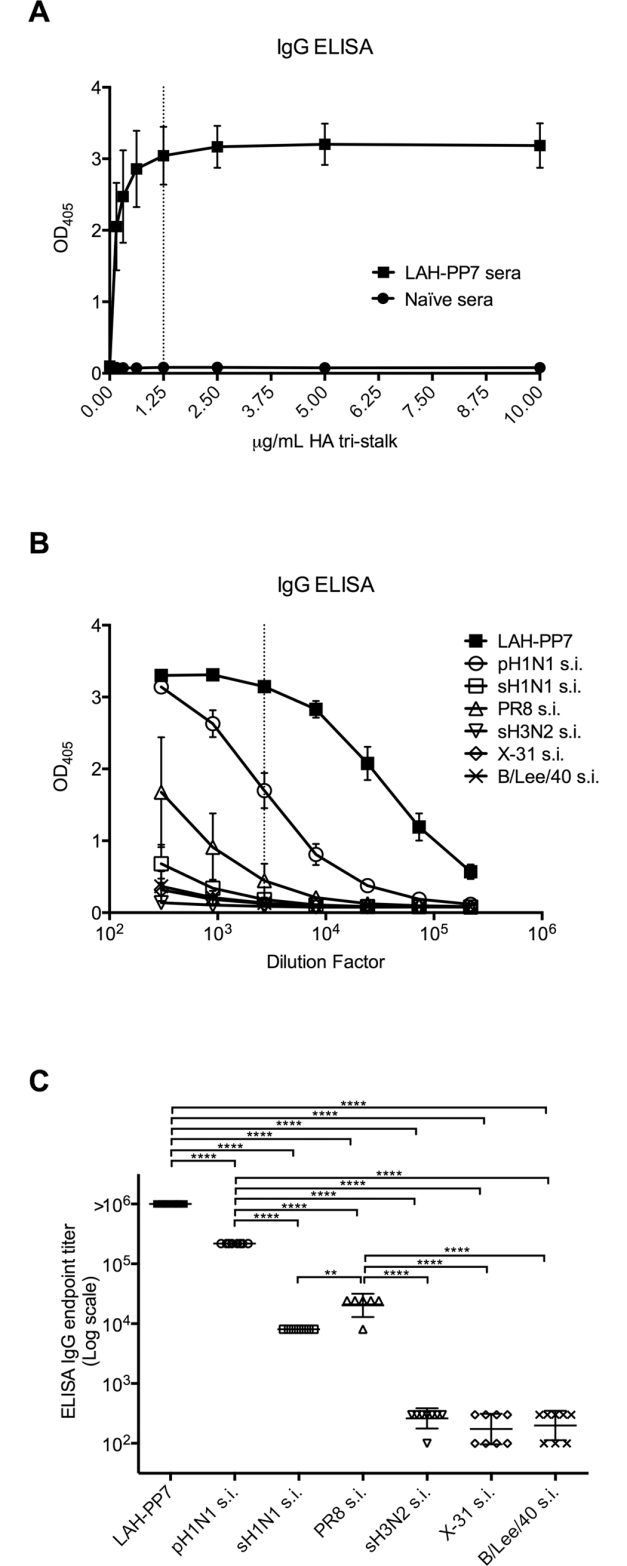
HA tri-stalk specific seroreactivity in mice. (A) Serial dilutions of HA tri-stalk protein (0.15625, 0.3125, 0.625, 1.25, 2.5, 5.0 and 10.0 μg/mL) tested against 1:2700 dilution of sera from LAH-PP7 vaccinated mice or naïve mice. (B) Total IgG ELISA was used to determine the HA tri-stalk specific seroreactivity induced by vaccination with LAH-PP7 or by sublethal infection (s.i.) of A/Luxembourg/46/2009 pandemic H1N1 (pH1N1), A/Texas/36/91 seasonal H1N1 (sH1N1), A/Puerto Rico/8/34 H1N1 (PR8), A/Lux/01/2005 seasonal H3N2 (sH3N2), A/Aïshi/68 H3N2 (X-31), or B/Lee/40 virus. Total HA tri-stalk specific IgG titers were assayed in sera after the third immunization with LAH-PP7 or after 2 weeks post sublethal virus infection. (C) Comparison of HA tri-stalk specific IgG endpoint titers (n = 8 mice/group; **p<0.001, ****p<0.00001).

In order to set up the optimal condition using HA tri-stalk protein as a coating antigen in indirect ELISA for the detection of seroreactivity to HA stem, the checkerboard titration method was conducted using sera from mice immunized with LAH-PP7 fusion protein (see Materials and methods) as positive samples and naïve mouse sera as negative controls. Optimal signal to noise (S/N) ratio was found with 1.25 μg/mL coated HA tri-stalk protein and serum dilution of 1:2700 (S/N ratio = 36, Fig 3A). The LAH-PP7 construct was originally designed to expose LAH peptide on the surface of bacteriophage PP7 shells in a form of natural trimer. Although we failed to detect the formation of VLPs, expression of this chimeric construct resulted in formation of soluble protein aggregates (S1 Fig) capable to induce strong seroconversion to both group 1 and group 2 HA antigens in mice (S2 Fig), which makes LAH-PP7 fusion protein particularly suitable to assess HA stem-specific Ab responses in mice.

These ELISA conditions were then used to determine the HA tri-stalk specific total IgG titer in sera (3-fold dilutions from 1:300, to 1:218700) from mice vaccinated with LAH-PP7 or sublethally infected (s.i.) by pH1N1, sH1N1, PR8, sH3N2, X-31, or B/Lee/40 virus (Fig 3B). As expected, vaccination with LAH-PP7 induced significantly higher level of HA tri-stalk specific total IgG comparing to sublethal infection by viruses of all the tested strains (Fig 3B and 3C). Nevertheless, mice s.i. by group 1 viruses pH1N1 or PR8 showed significantly higher levels of HA tri-stalk specific IgG than the animals s.i. by the other viruses (Fig 3C). Mice s.i. by strains with group 2 (sH3N2 and X-31) or B/Lee/40 virus exhibited no seroreactivity with HA tri-stalk protein (Fig 3C).

### HA tri-stalk reactive Ab-secreting cells in mouse splenocytes after vaccination

**Fig 4:**
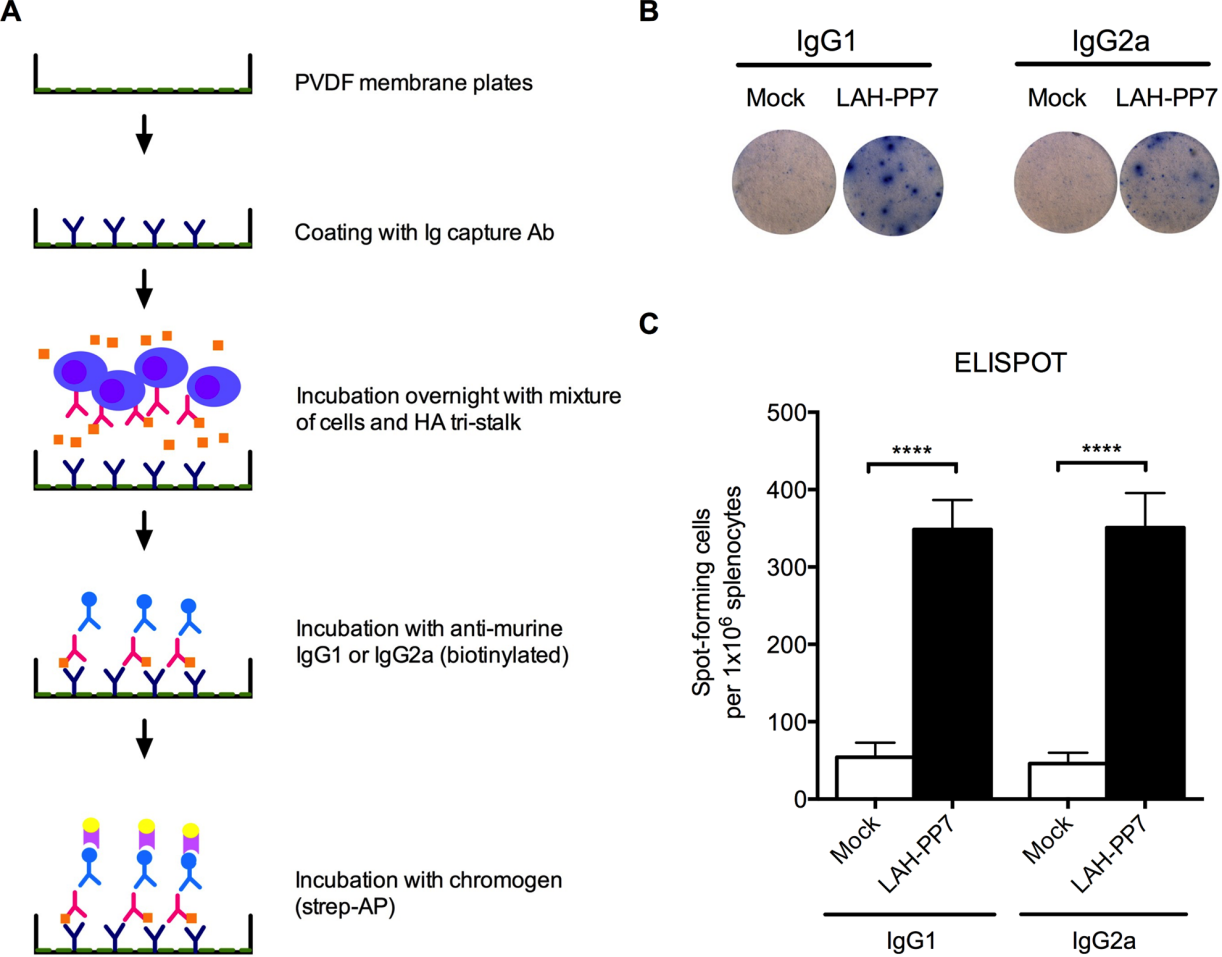
Detection of HA tri-stalk specific memory B cells. (A) ELISPOT assay workflow. (B) Representative pictures of spot-forming IgG1- or IgG2a-secreting cells in mice injected with PBS (mock) or LAH-PP7 fusion protein. (C) Comparison of total IgG1- and IgG2a-secreting B cells under stimulation by HA tri-stalk protein in the spleen of mice from mock and LAH-PP7 vaccinated groups (n = 8 mice/group; [29], ****p<0.00001).

To evaluate HA tri-stalk specific Ab-secreting B cells induced by vaccination, BALB/c mice were immunized 3 times i.p. with LAH-PP7 fusion protein that induced high levels of humoral immunity against LAH domain of HA stem as described above. Two weeks after the last immunization, the splenocytes were stimulated *ex vivo* with HA tri-stalk protein. HA tri-stalk reactive Ab-secreting cells were enumerated by ELISPOT assay (Fig 4A). Visually distinct populations of IgG1- and IgG2a- secreting B cells were detected after HA tri-stalk protein stimulation in the vaccinated but not in the mock mice. Although in LAH-PP7 vaccinated mice the IgG1-secreting cells formed larger immunospots than the IgG2a-secreting cells (Fig 4B), there was no significant difference in absolute numbers between both isotype subclasses (Fig 4C). The vaccinated mice showed significantly (up to 10 times) higher numbers of both IgG1- and IgG2a-secreting cells after HA tri-stalk immunization than the mock mice (Fig 4C). This further supports the value of HA tri-stalk protein for monitoring vaccine-induced memory B cell responses against the stem domain.

### Pre-pandemic HA tri-stalk specific Abs in SW

**Fig 5:**
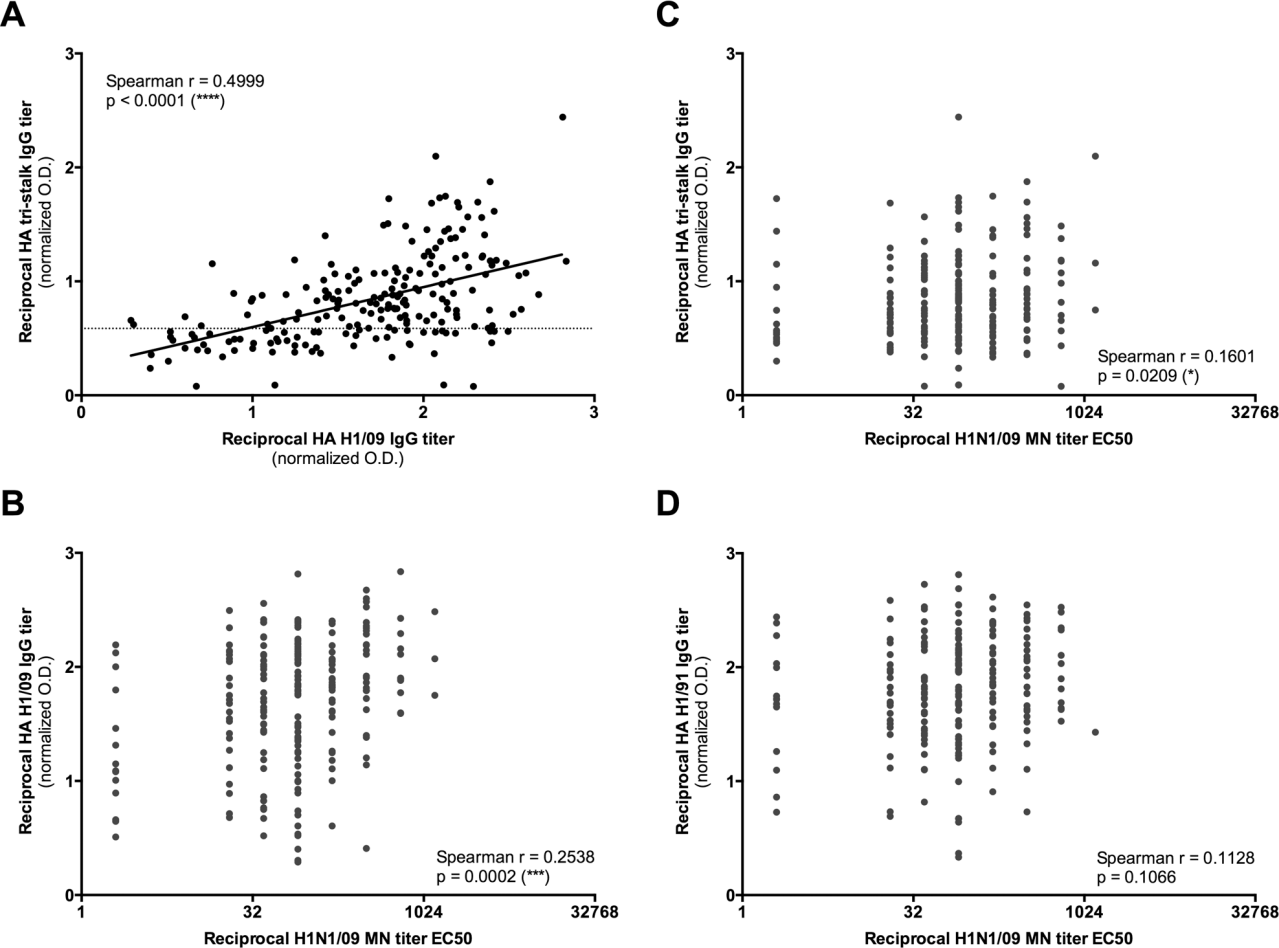
HA tri-stalk specific Abs and neutralizing Abs against pandemic H1N1/09 virus in human. (A) Correlations between HA tri-stalk specific Abs and HA H1/09 specific Abs. Sera with an OD >0.5868 (dotted line) were classified as seropositive to HA tri-stalk. (B) Correlations between H1N1/09 microneutralization Abs and Abs to HA H1/09. (C) Correlations between H1N1/09 microneutralization Abs and HA tri-stalk specific Abs. (D) Correlations between H1N1/09 microneutralization Abs and Abs to HA H1/91 (A/Texas/36/1991).

The newly developed indirect ELISA using the purified HA tri-stalk protein as a coating antigen was further applied to examine human seroreactivity to HA stem (as described in the Materials and methods). The mean OD of the negative sera was found 0.2115, +/- 0.1251 (SD), and the cut-off point of 0.5868 was calculated as the mean of the negative control sera plus three SD. Sera with an OD >0.5868 were classified as seropositive. A total of 147 of 211 (70%) pre-pandemic SW sera were considered positive for HA stem-specific Abs, whereas only 30 of 71 (42%) pre-pandemic sera from non-SW showed positive for HA stem-specific Abs (S3 Fig). Interestingly, there was a strong correlation between HA stem-specific IgG titer and HA H1/09 IgG titer in these pre-pandemic sera (Fig 5A).

We observed a stronger correlation between HA tri-stalk specific IgG titer and HA H1/09 IgG titer (Fig 5A, Spearman r = 0.4999, p < 10^−4^). These results indicate that already before the pandemic (H1N1) 2009 virus had spread to Western Europe, substantial levels of cross-reactive Abs against HA stem and HA H1/09 could be detected in humans with professional contact to swine.

Furthermore, we tested the SW for neutralizing Abs against influenza A/Luxembourg/43/2009 (H1N1/09) virus. Significant correlations were observed between H1N1/09 neutralizing titers with HA H1/09 IgG titer (Fig 5B, Spearman r = 0.2538, p = 0.0002) as well as with HA stem-specific IgG titer (Fig 5C, Spearman r = 0.1601, p = 0.0209). Whereas, IgG levels to HA of a pre-existing seasonal H1N1 virus, A/Texas/36/1991, showed no correlation with H1N1/09 neutralizing titers (Fig 5D, Spearman r = 0.1128, p = 0.1066). These observations in SW indicate that pre-pandemic Ab levels to HA tri-stalk protein also translated into neutralizing activities against H1N1/09 virus.

## Discussion

In this study, we have focused on the structure and applications of a novel HA tri-stalk protein comprising LAH region which has attracted much attention as a potential universal influenza vaccine component [16–19]. *E*. *coli* expressed tri-stalk protein can be purified to near homogeneity using a convenient three-step chromatography method. Structural data revealed that tri-stalk protein folding closely matches the native HA post-fusion conformation. This is in line with a previous study where HA2 polypeptide 38–175 was also *E*. *coli* expressed in post-fusion form [30]. Our tri-stalk protein is significantly shorter and corresponds to HA2 amino acids 59–130. In contrast to polypeptide 38–175 being able to resist incubation at 68°C some portion of tri-stalk protein was found unassembled already after heating at 55°C indicating that the lack of internal cysteines negatively affects its thermostability (Fig 1C). Nevertheless, the outcome of assembled tri-stalk protein using our purification method is estimated at about 5 mg/g of wet cells which makes it attractive for downstream immunogenicity and antigenicity studies and co-crystallization for structural and immunological analysis.

Since the discovery of broadly neutralizing Abs against HA stem [8] a number of attempts have been performed to construct universal stem-based influenza vaccines. One direction of investigations is targeted to design the trimeric mini-stem constructs being able to bind conformational Abs and protect mice from lethal influenza virus challenge [31–32]. The constructs described are notably longer than tri-stalk protein and contain either intermolecular disulfide bridge, or trimerization (foldon) motif to stabilize the structure. At this stage, our aim was not to outperform these proteins in terms of protection, but to produce an alternative variant in a cost-effective way to be applied in several immune assays for evaluating the future universal vaccine-induced response. While tri-stalk protein is only 72 aa long, it is promising candidate for exposition on the surface of VLPs which is a highly powerful platform for vaccine construction. Incorporation of the LAH within the major immunodominant loop of the hepatitis B core protein allows formation of chimeric VLPs [17–19], however, anchoring of both LAH ends prevents folding of native trimeric conformation. Nevertheless, this strategy led to strong seroconversion to both HA group antigens in mice [18] while immunization with tri-stalk protein alone generated Abs only against homologous group HA antigen (S4 Fig). Ideally, both approaches should be combined to obtain possible synergistic effect. Genetic fusion of LAH-encoding sequence with N- or C-termini of a carrier protein gene would theoretically allow such assembly. Among large variety of carriers coat proteins of small RNA phages organized in simple *T* = 3 symmetry particles offer an attractive scaffold for exposition of trimeric proteins in their native form, since N- and C- terminal ends of the phage coat protein are arranged closely around quasi-threefold axis of *T* = 3 particle. Although we failed to expose LAH peptide on the surface of PP7 VLPs (S1 Fig), it could be speculated that this chimeric protein at least partially assembles in trimeric structures capable to elicit potent immune responses (S2 Fig). This assembly is, however, hard to prove due to irregularity of LAH-VLP aggregates (S1 Fig).

Although HA stem is closely packed to the viral membrane and therefore partly covered by the head domain, the majority of conserved region is accessible to anti-stem Abs [33]. However, immune responses post-infection and post-vaccination are mainly targeted to the immunodominant HA head region [11]. Accordingly, it is well established that anti-stem Abs are normally not found in individuals vaccinated with seasonal inactivated influenza virus vaccines whereas low levels of these Abs are induced by natural virus infection in humans as well as in mice [34]. In contrast, infection of individuals with 2009 pandemic H1N1 virus induced high levels of stem-reactive Ab responses [35]. Cross-reactive immunity derived from anti-stem-specific Abs also tends to increase upon exposure to antigenically diverse influenza viruses [36], [8]. Swine play a particular role in cross-species transmission of diverse influenza viruses because they are susceptible to both avian and human influenza viruses and can act as “mixing vessel” for reassortment of viral gene segments [37]. Previous study showed that, before the outbreak of the pandemic H1N1 2009, persons with professional contact with swine obtained higher serological cross-reactivity with various influenza viruses, including pandemic H1N1 2009 virus, European avian-like subtype H1N1 SIV, and 2007–2008 seasonal influenza subtype H1N1 virus [20]. We therefore hypothesize that these SW harbor pre-existing cross-immunity against various influenza strains, possibly mediated by Ab targeting to conserved viral epitopes, like the HA stem. Indeed, using tri-stalk protein based ELISA to determine the binding of the cross-reactive Abs in these pre-pandemic sera, the results show that Abs targeting the tri-stalk epitope significantly correlate with the Abs specific to the novel antigen, HA H1/09. In addition, tri-stalk specific Abs also have a positive correlation with the neutralizing activities against the pandemic H1N1 2009 virus.

## Conclusion

Altogether, our results demonstrate that the novel HA tri-stalk protein, which comprises of the HA LAH region, can be applied in various immunological assays to determine HA stem-specific humoral responses in both clinical and animal samples. The conserved feature of LAH domain makes the HA tri-stalk protein a promising tool for evaluation of pre-existing or cross-reactive immunity to a newly emerged influenza viruses.

## Supporting information

S1 Fig Purification of LAH-PP7 fusion protein. (A) Coomassie-stained PAAG illustrating individual purification steps. Lane 1, summary expression; lane 2, supernatant; lane 3, urea extract loaded onto Superdex column; lane 4, peak protein fraction from Superdex column. (B) Size-exclusion chromatography profile and (C) Electron microscopy indicating aggregation of target protein.
(PDF)

S2 Fig The cross-reactivity of mouse sera. (A) Reactivity with group 1 HA proteins (H1, H2, H5, H9, H11 and H16). (B) Reactivity with group 2 HA proteins (H3, H4, H7, H10, H14 and H15). Mice were immunized with LAH-PP7 fusion protein or adjuvant alone (mock). The panels show the mean and standard deviation of the optical density at 405 nm of the sera from 10 mice per group.
(PDF)

S3 Fig Comparison of HA tri-stalk ELISA results between SW and non-SW. HA tri-stalk specific seroreactivities were evaluated in pre-pandemic sera from 211 SW and 71 non-SW.
(TIFF)

S4 Fig Comparison of Ab responses after vaccination of LAH-HBc VLPs [18] and tri-stalk protein. (TIFF)

S5 Fig Full wwPDB X-ray structure validation report. (PDF)

S6 Fig The ARRIVE guidelines checklist. (DOCX)

## References

[1] Preaud E, Durand L, Macabeo B, Farkas N, Sloesen B, Palache A, et al Annual public health and economic benefits of seasonal influenza vaccination: a European estimate. BMC Public Health. 2014;14:813 10.1186/1471-2458-14-813 25103091 PMC4141103

[2] Paules C, Subbarao K. Influenza. Lancet. 2017;390(10095):697–708. 10.1016/S0140-6736(17)30129-0 28302313

[3] de Vries RD, Herfst S, Richard M. Avian Influenza A Virus Pandemic Preparedness and Vaccine Development. Vaccines (Basel). 2018;6(3). 10.3390/vaccines6030046 30044370 PMC6161001

[4] Johnson NP, Mueller J. Updating the accounts: global mortality of the 1918–1920 "Spanish" influenza pandemic. Bull Hist Med. 2002;76(1):105–15. . 11875246 10.1353/bhm.2002.0022

[5] Yuen KY, Chan PK, Peiris M, Tsang DN, Que TL, Shortridge KF, et al Clinical features and rapid viral diagnosis of human disease associated with avian influenza A H5N1 virus. Lancet. 1998;351(9101):467–71. . 9482437 10.1016/s0140-6736(98)01182-9

[6] Gao R, Cao B, Hu Y, Feng Z, Wang D, Hu W, et al Human infection with a novel avian-origin influenza A (H7N9) virus. N Engl J Med. 2013;368(20):1888–97. 10.1056/NEJMoa1304459 23577628

[7] Wei SH, Yang JR, Wu HS, Chang MC, Lin JS, Lin CY, et al Human infection with avian influenza A H6N1 virus: an epidemiological analysis. Lancet Respir Med. 2013;1(10):771–8. 10.1016/S2213-2600(13)70221-2 24461756 PMC7164810

[8] Krammer F, Palese P. Influenza virus hemagglutinin stalk-based antibodies and vaccines. Curr Opin Virol. 2013;3(5):521–30. 10.1016/j.coviro.2013.07.007 23978327 PMC3804342

[9] Wilson IA, Skehel JJ, Wiley DC. Structure of the haemagglutinin membrane glycoprotein of influenza virus at 3 A resolution. Nature. 1981;289(5796):366–73. . 7464906 10.1038/289366a0

[10] Xu R, Wilson IA. Structural characterization of an early fusion intermediate of influenza virus hemagglutinin. J Virol. 2011;85(10):5172–82. 10.1128/JVI.02430-10 21367895 PMC3126156

[11] Ellebedy AH, Ahmed R. Re-engaging cross-reactive memory B cells: the influenza puzzle. Front Immunol. 2012;3:53 10.3389/fimmu.2012.00053 22566934 PMC3342361

[12] Heaton NS, Sachs D, Chen CJ, Hai R, Palese P. Genome-wide mutagenesis of influenza virus reveals unique plasticity of the hemagglutinin and NS1 proteins. Proc Natl Acad Sci U S A. 2013;110(50):20248–53. 10.1073/pnas.1320524110 24277853 PMC3864309

[13] Neu KE, Henry Dunand CJ, Wilson PC. Heads, stalks and everything else: how can antibodies eradicate influenza as a human disease? Curr Opin Immunol. 2016;42:48–55. 10.1016/j.coi.2016.05.012 27268395 PMC5086271

[14] Kallewaard NL, Corti D, Collins PJ, Neu U, McAuliffe JM, Benjamin E, et al Structure and Function Analysis of an Antibody Recognizing All Influenza A Subtypes. Cell. 2016;166(3):596–608. 10.1016/j.cell.2016.05.073 27453466 PMC4967455

[15] Dreyfus C, Laursen NS, Kwaks T, Zuijdgeest D, Khayat R, Ekiert DC, et al Highly conserved protective epitopes on influenza B viruses. Science. 2012;337(6100):1343–8. 10.1126/science.1222908 22878502 PMC3538841

[16] Wang TT, Tan GS, Hai R, Pica N, Ngai L, Ekiert DC, et al Vaccination with a synthetic peptide from the influenza virus hemagglutinin provides protection against distinct viral subtypes. Proc Natl Acad Sci U S A. 2010;107(44):18979–84. 10.1073/pnas.1013387107 20956293 PMC2973924

[17] Chen S, Zheng D, Li C, Zhang W, Xu W, Liu X, et al Protection against multiple subtypes of influenza viruses by virus-like particle vaccines based on a hemagglutinin conserved epitope. Biomed Res Int. 2015;2015:901817 10.1155/2015/901817 25767809 PMC4341857

[18] Kazaks A, Lu IN, Farinelle S, Ramirez A, Crescente V, Blaha B, et al Production and purification of chimeric HBc virus-like particles carrying influenza virus LAH domain as vaccine candidates. BMC Biotechnol. 2017;17(1):79 10.1186/s12896-017-0396-8 29126399 PMC5681787

[19] Ramirez A, Morris S, Maucourant S, D'Ascanio I, Crescente V, Lu IN, et al A virus-like particle vaccine candidate for influenza A virus based on multiple conserved antigens presented on hepatitis B tandem core particles. Vaccine. 2018;36(6):873–80. 10.1016/j.vaccine.2017.12.053 29306508

[20] Gerloff NA, Kremer JR, Charpentier E, Sausy A, Olinger CM, Weicherding P, et al Swine influenza virus antibodies in humans, western Europe, 2009. Emerg Infect Dis. 2011;17(3):403–11. 10.3201/eid1703.100851 21392430 PMC3165996

[21] Battye TG, Kontogiannis L, Johnson O, Powell HR, Leslie AG. iMOSFLM: a new graphical interface for diffraction-image processing with MOSFLM. Acta Crystallogr D Biol Crystallogr. 2011;67(Pt 4):271–81. 10.1107/S0907444910048675 21460445 PMC3069742

[22] Scaling Evans P. and assessment of data quality. Acta Crystallogr D Biol Crystallogr. 2006;62(Pt 1):72–82. 16369096 10.1107/S0907444905036693

[23] Potterton E, Briggs P, Turkenburg M, Dodson E. A graphical user interface to the CCP4 program suite. Acta Crystallogr D Biol Crystallogr. 2003;59(Pt 7):1131–7. . 12832755 10.1107/s0907444903008126

[24] McCoy AJ, Grosse-Kunstleve RW, Storoni LC, Read RJ. Likelihood-enhanced fast translation functions. Acta Crystallogr D Biol Crystallogr. 2005;61(Pt 4):458–64. 10.1107/S0907444905001617 15805601

[25] Bullough PA, Hughson FM, Skehel JJ, Wiley DC. Structure of influenza haemagglutinin at the pH of membrane fusion. Nature. 1994;371(6492):37–43. 10.1038/371037a0 8072525

[26] Cowtan K. The Buccaneer software for automated model building. 1. Tracing protein chains. Acta Crystallogr D Biol Crystallogr. 2006;62(Pt 9):1002–11. 10.1107/S0907444906022116 16929101

[27] Emsley P, Cowtan K. Coot: model-building tools for molecular graphics. Acta Crystallogr D Biol Crystallogr. 2004;60(Pt 12 Pt 1):2126–32. 10.1107/S0907444904019158 15572765

[28] Murshudov GN, Skubak P, Lebedev AA, Pannu NS, Steiner RA, Nicholls RA, et al REFMAC5 for the refinement of macromolecular crystal structures. Acta Crystallogr D Biol Crystallogr. 2011;67(Pt 4):355–67. 10.1107/S0907444911001314 21460454 PMC3069751

[29] Sasaki S, Sullivan M, Narvaez CF, Holmes TH, Furman D, Zheng NY, et al Limited efficacy of inactivated influenza vaccine in elderly individuals is associated with decreased production of vaccine-specific antibodies. J Clin Invest. 2011;121(8):3109–19. 10.1172/JCI57834 21785218 PMC3148747

[30] Chen J, Wharton SA, Weissenhorn W, Calder LJ, Hughson FM, Skehel JJ, et al A soluble domain of the membrane-anchoring chain of influenza virus hemagglutinin (HA2) folds in Escherichia coli into the low-pH-induced conformation. Proc Natl Acad Sci U S A. 1995;92(26):12205–9. . 8618870 10.1073/pnas.92.26.12205 PMC40325

[31] Valkenburg SA, Mallajosyula VV, Li OT, Chin AW, Carnell G, Temperton N, et al Stalking influenza by vaccination with pre-fusion headless HA mini-stem. Sci Rep. 2016;6:22666 10.1038/srep22666 26947245 PMC4780079

[32] Impagliazzo A, Milder F, Kuipers H, Wagner MV, Zhu X, Hoffman RM, et al A stable trimeric influenza hemagglutinin stem as a broadly protective immunogen. Science. 2015;349(6254):1301–6. 10.1126/science.aac7263 26303961

[33] Harris AK, Meyerson JR, Matsuoka Y, Kuybeda O, Moran A, Bliss D, et al Structure and accessibility of HA trimers on intact 2009 H1N1 pandemic influenza virus to stem region-specific neutralizing antibodies. Proc Natl Acad Sci U S A. 2013;110(12):4592–7. 10.1073/pnas.1214913110 23460696 PMC3607006

[34] Margine I, Hai R, Albrecht RA, Obermoser G, Harrod AC, Banchereau J, et al H3N2 influenza virus infection induces broadly reactive hemagglutinin stalk antibodies in humans and mice. J Virol. 2013;87(8):4728–37. 10.1128/JVI.03509-12 23408625 PMC3624338

[35] Pica N, Hai R, Krammer F, Wang TT, Maamary J, Eggink D, et al Hemagglutinin stalk antibodies elicited by the 2009 pandemic influenza virus as a mechanism for the extinction of seasonal H1N1 viruses. Proc Natl Acad Sci U S A. 2012;109(7):2573–8. 10.1073/pnas.1200039109 22308500 PMC3289326

[36] Miller MS, Gardner TJ, Krammer F, Aguado LC, Tortorella D, Basler CF, et al Neutralizing antibodies against previously encountered influenza virus strains increase over time: a longitudinal analysis. Sci Transl Med. 2013;5(198):198ra07. 10.1126/scitranslmed.3006637 23946196 PMC4091683

[37] Shortridge KF, Webster RG, Butterfield WK, Campbell CH. Persistence of Hong Kong influenza virus variants in pigs. Science. 1977;196(4297):1454–5. . 867041 10.1126/science.867041

