# Supplementary figures and images for: Structure and applications of novel influenza HA tri-stalk protein for evaluation of HA stem-specific immunity
Source: PLoS One. 2018 Sep 27;13(9):e0204776. doi: 10.1371/journal.pone.0204776 (PMC6160157; doi:10.1371/journal.pone.0204776)

**A**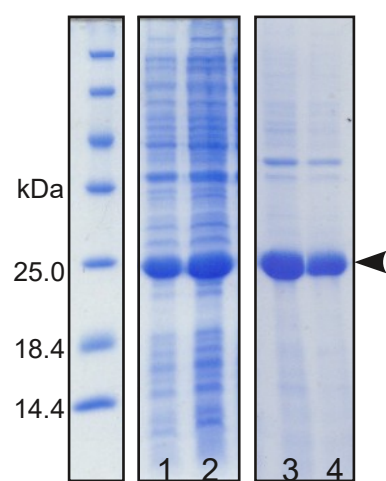**B**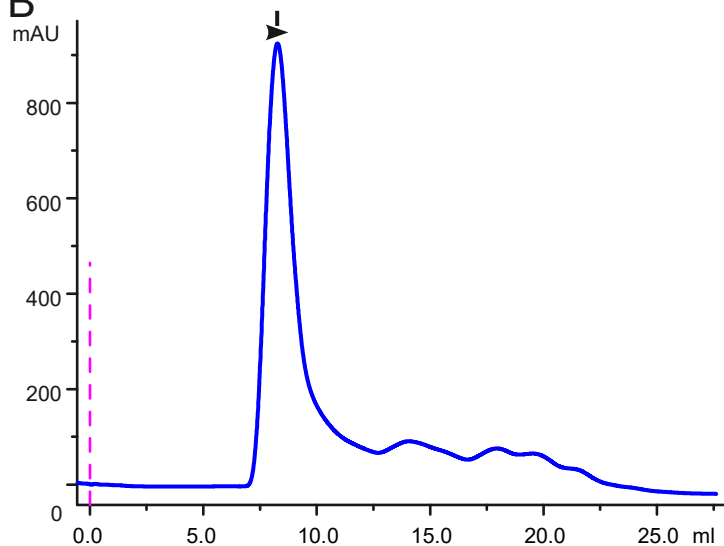**C**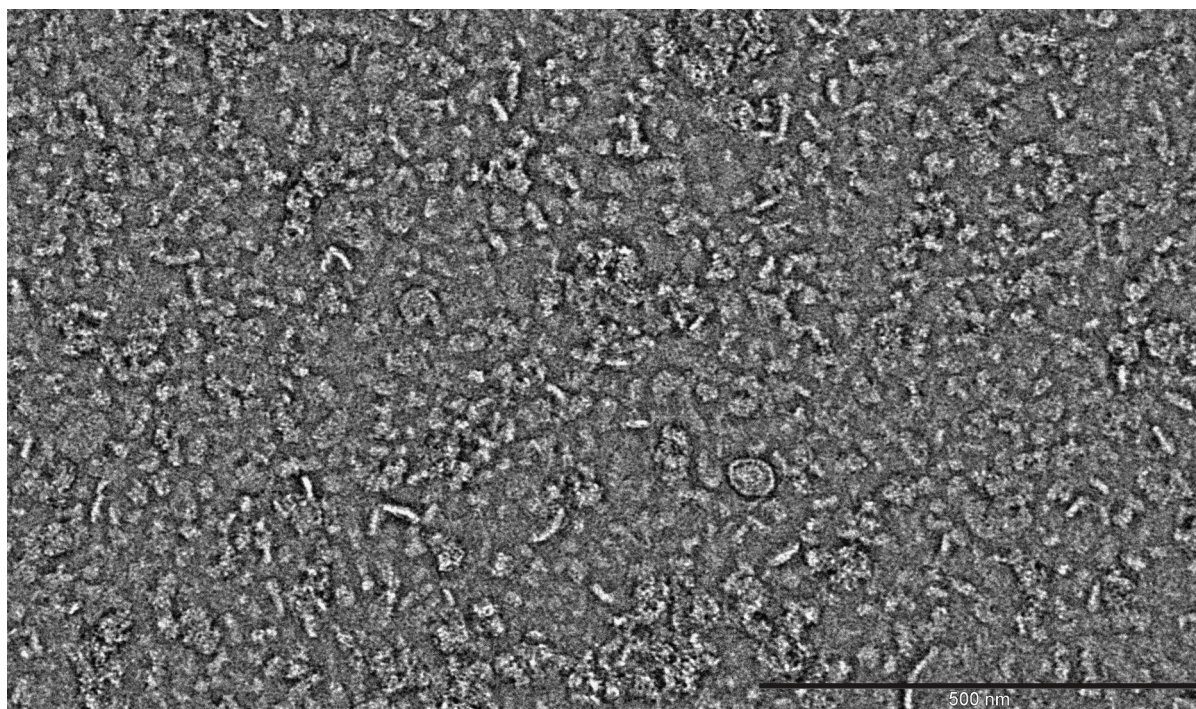

Supplement: S1 Fig — (A) Coomassie-stained PAAG illustrating individual purification steps. Lane 1, summary expression; lane 2, supernatant; lane 3, urea extract loaded onto Superdex column; lane 4, peak protein fraction from Superdex column. (B) Size-exclusion chromatography profile and (C) Electron microscopy indicating aggregation of target protein. (PDF) [file pone.0204776.s001.pdf]

**A**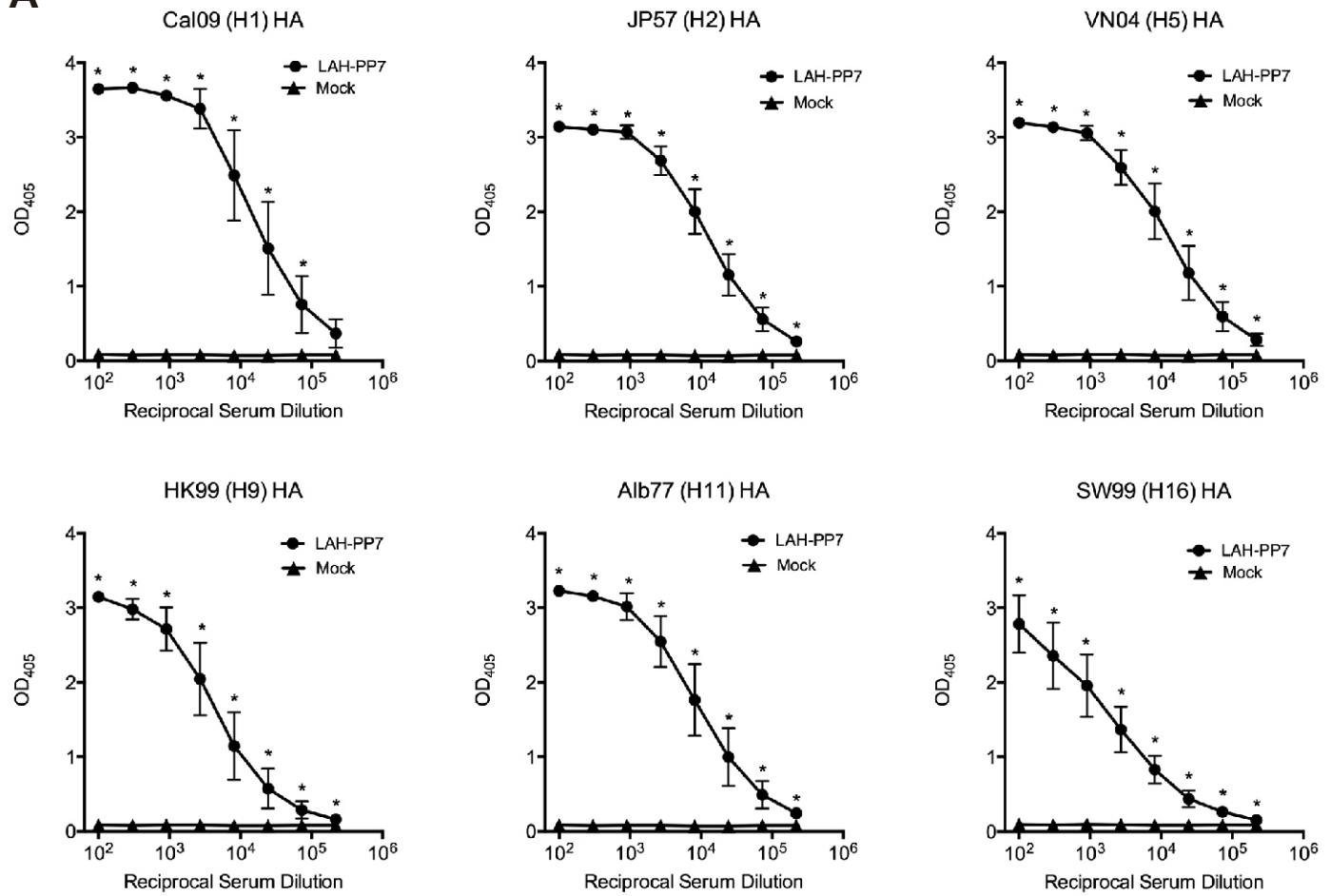**B**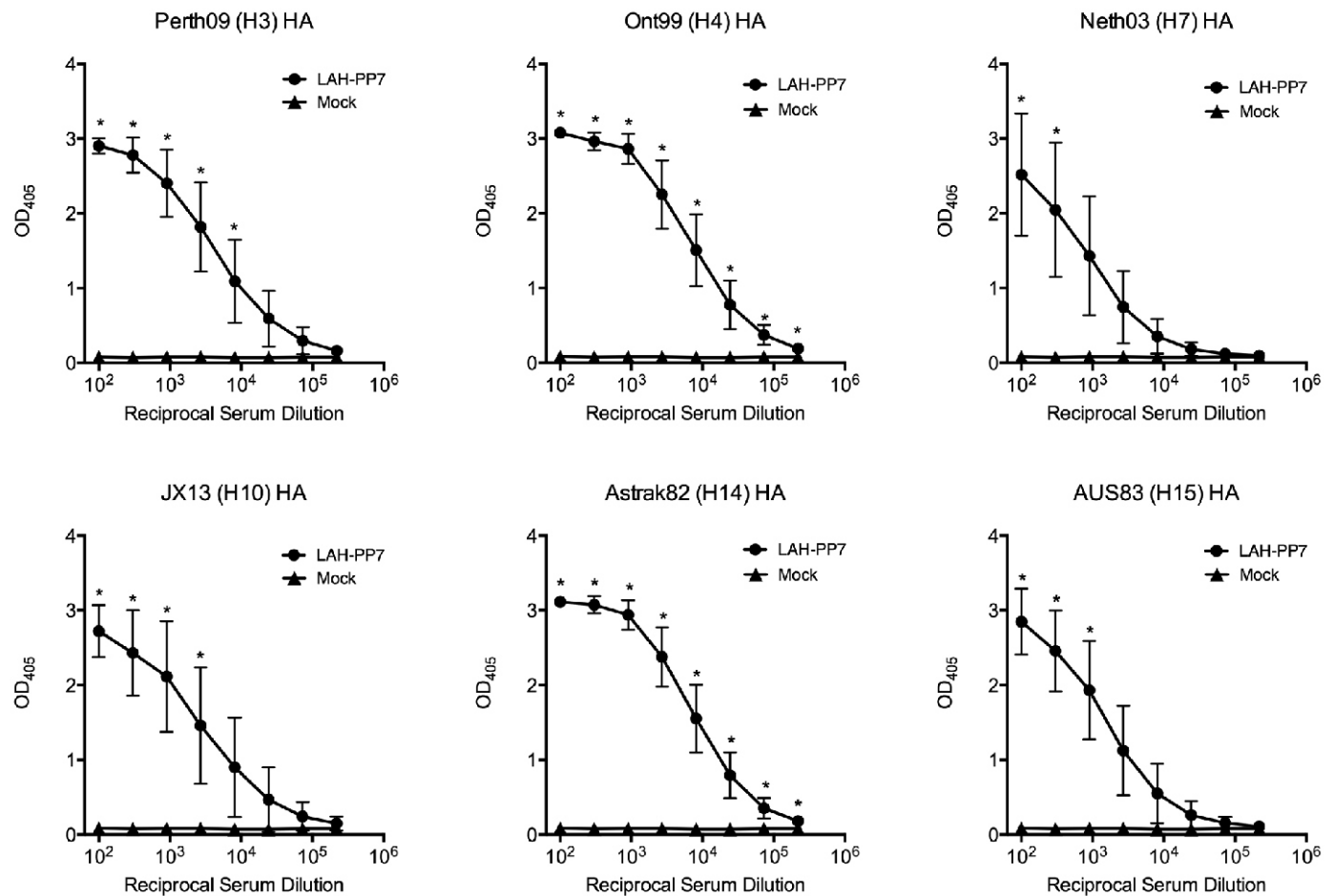

Supplement: S2 Fig — (A) Reactivity with group 1 HA proteins (H1, H2, H5, H9, H11 and H16). (B) Reactivity with group 2 HA proteins (H3, H4, H7, H10, H14 and H15). Mice were immunized with LAH-PP7 fusion protein or adjuvant alone (mock). The panels show the mean and standard deviation of the optical density at 405 nm of the sera from 10 mice per group. (PDF) [file pone.0204776.s002.pdf]

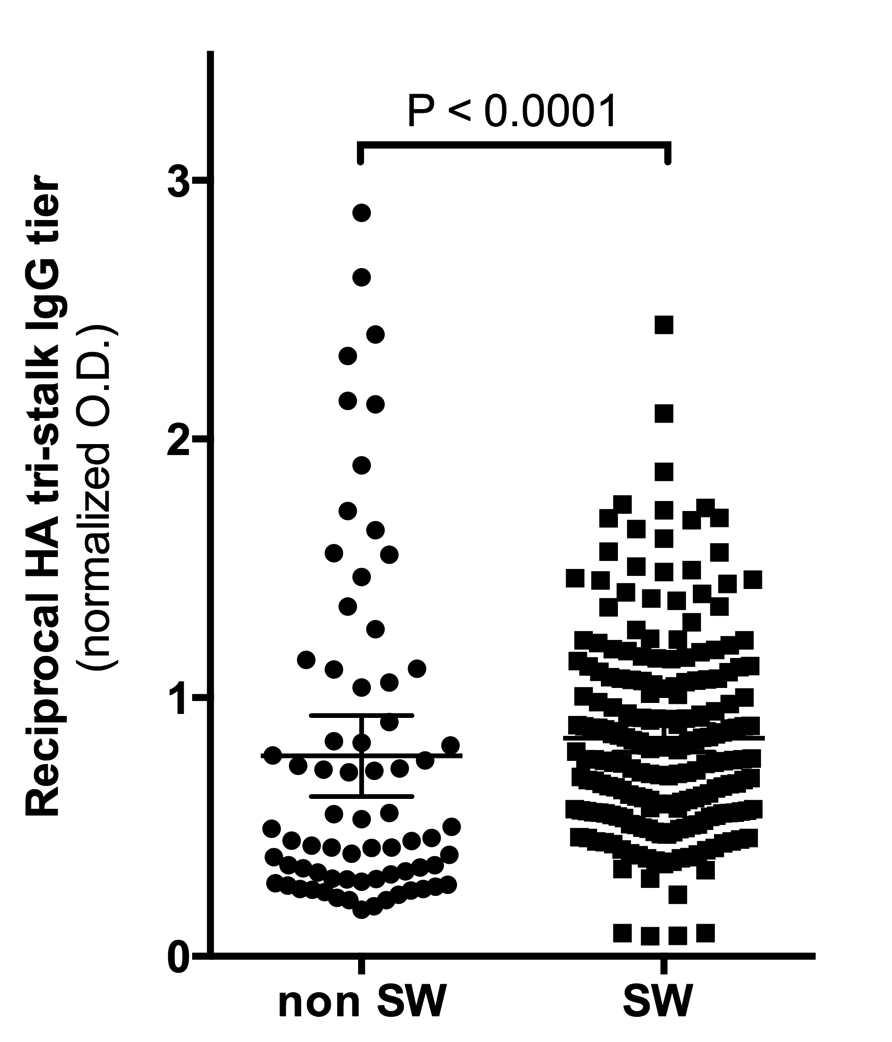

Supplement: S3 Fig — HA tri-stalk specific seroreactivities were evaluated in pre-pandemic sera from 211 SW and 71 non-SW. (TIFF) [file pone.0204776.s003.tiff]

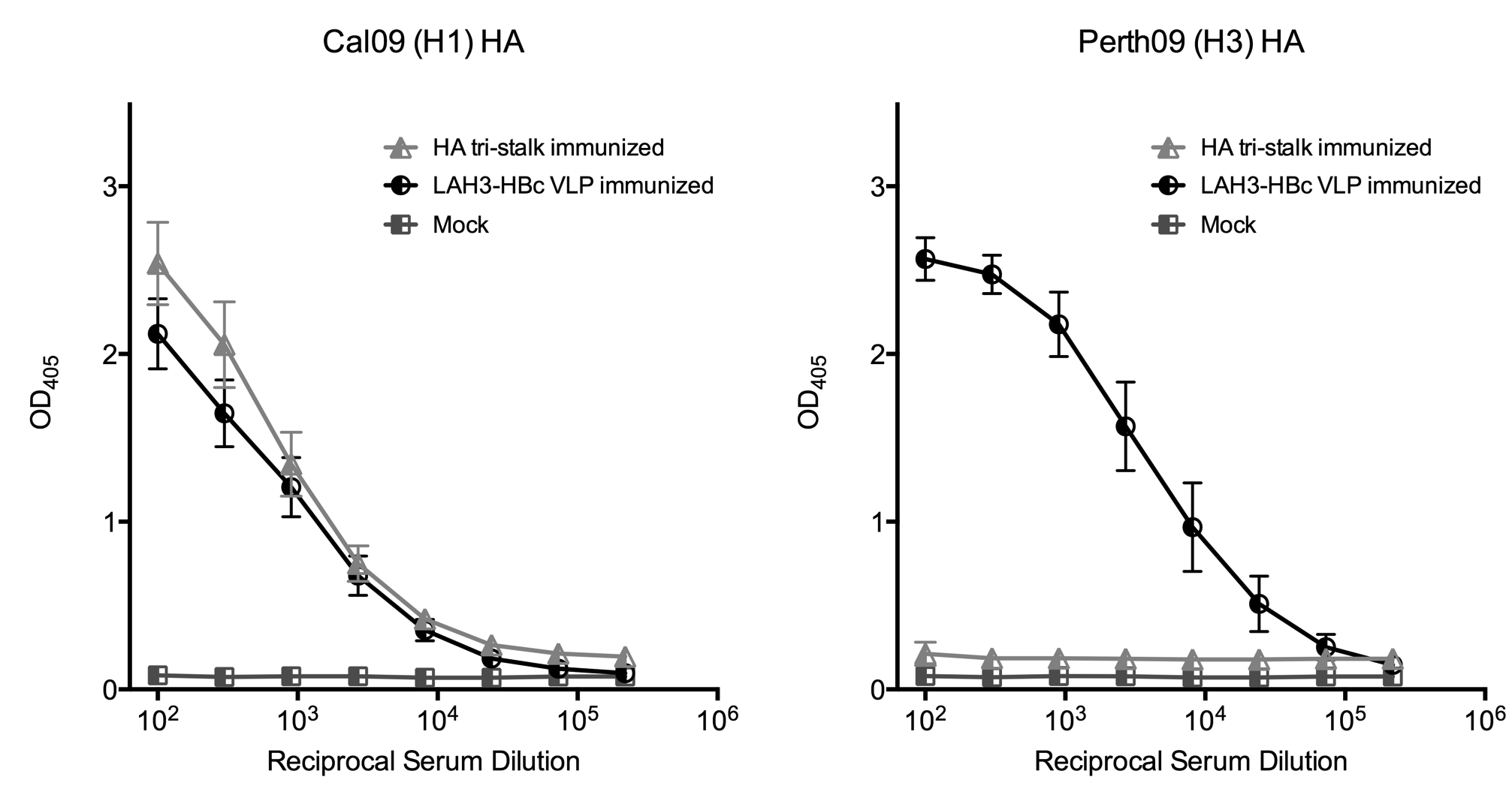

Supplement: S4 Fig — (TIFF) [file pone.0204776.s004.tiff]
