# Supplementary material for: Structure and applications of novel influenza HA tri-stalk protein for evaluation of HA stem-specific immunity
Source: PLoS One. 2018 Sep 27;13(9):e0204776. doi: 10.1371/journal.pone.0204776 (PMC6160157; doi:10.1371/journal.pone.0204776)

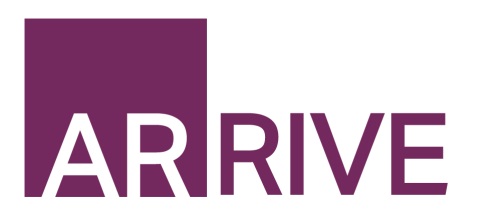


The ARRIVE Guidelines Checklist

Animal Research: Reporting In Vivo Experiments

Carol Kilkenny^1^, William J Browne^2^, Innes C Cuthill^3^, Michael Emerson^4^ and Douglas G Altman^5^

*^1^The National Centre for the Replacement, Refinement and Reduction of Animals in Research, London, UK, ^2^School of Veterinary Science, University of Bristol, Bristol, UK, ^3^School of Biological Sciences, University of Bristol, Bristol, UK, ^4^National Heart and Lung Institute, Imperial College London, UK, ^5^Centre for Statistics in Medicine, University of Oxford, Oxford, UK.*

|  | | ITEM | RECOMMENDATION | Section/ Paragraph |
| --- | --- | --- | --- | --- |
| 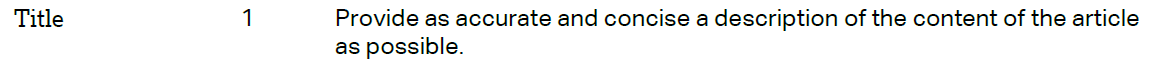 | | | Title |  |
| 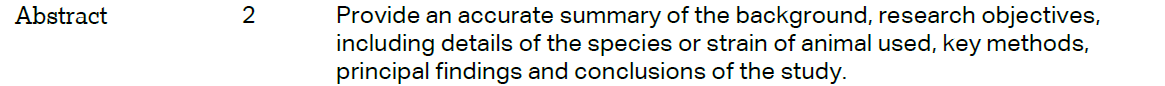 | | | Abstract |  |
| INTRODUCTION | | |  |  |
| 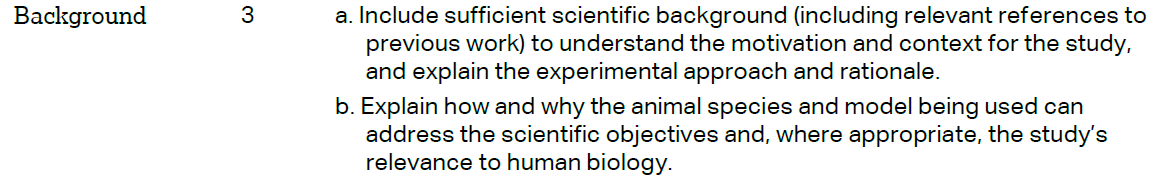 | | | Paragraphs 1-3  Paragraph 4 |  |
| 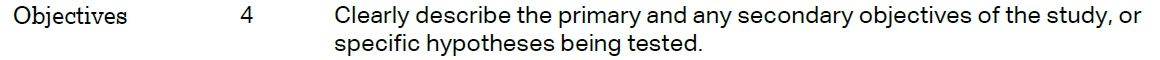 | | | Paragraph 4 |  |
| METHODS | | |  |  |
| 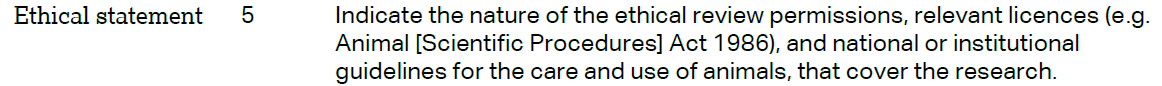 | | | Chapter 3,  Paragraph 1 |  |
| 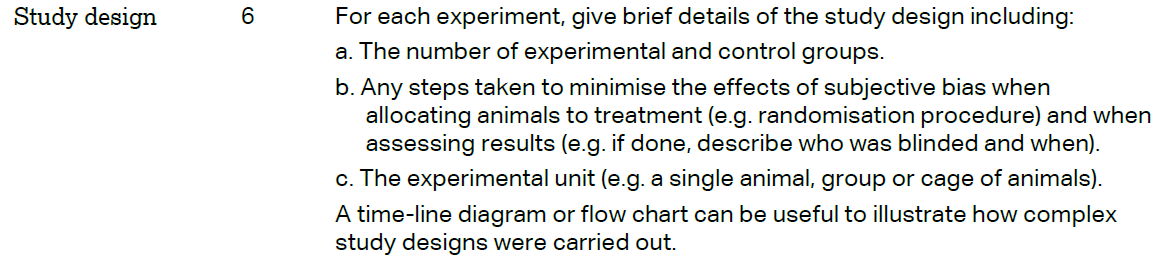 | | | Chapter 3, Paragraphs 2, 4  Chapter 3, Paragraph 1 |  |
| 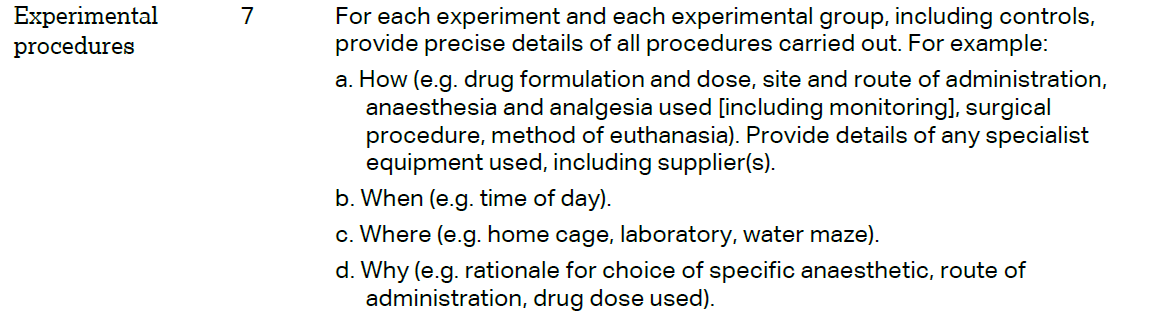 | | | Chapter 3,  Paragraphs 1, 2, 4 |  |
| 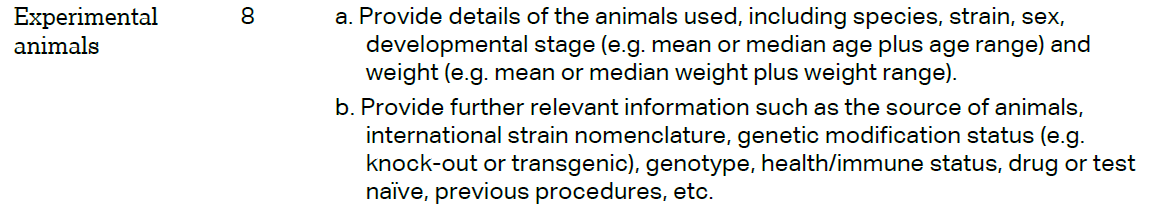 | | | Chapter 3, Paragraph 1 |  |

The ARRIVE guidelines. Originally published in *PLoS Biology*, June 2010^1^

| 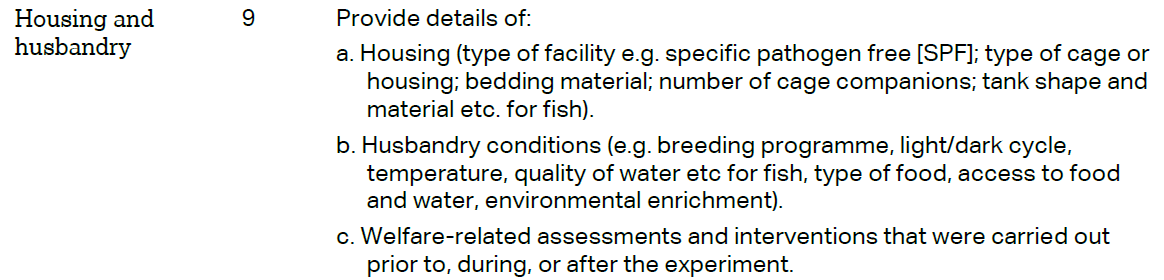 | Chapter 3, Paragraph 1 | |
| --- | --- | --- |
| 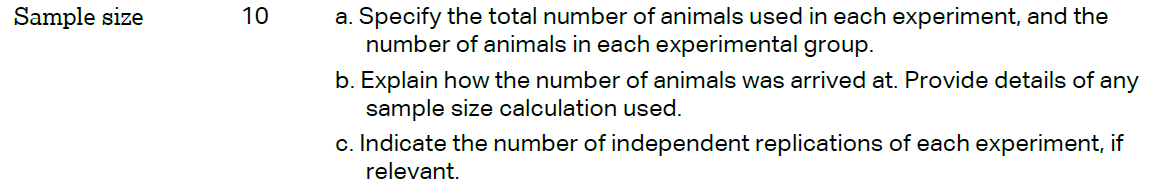 | Chapter 3, Paragraphs 2, 4 | |
| 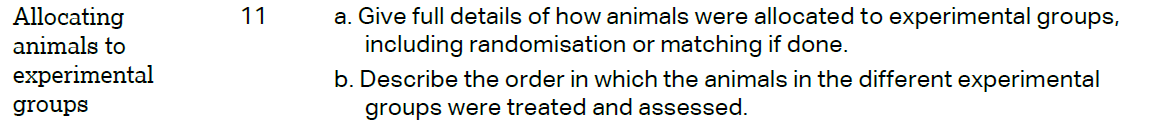 | Chapter 3, Paragraphs 2, 4 | |
| 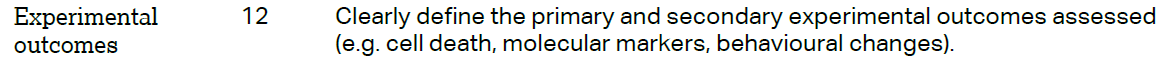 | Chapter 3, Paragraph 2 | |
| 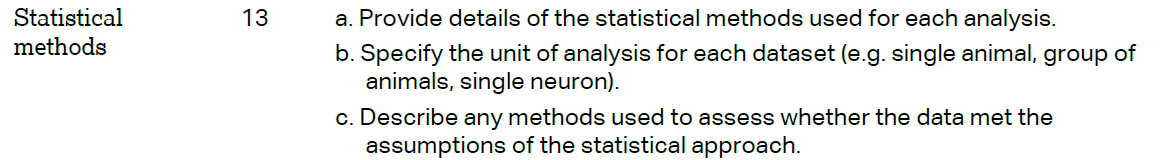 | Chapter 8 | |
| RESULTS |  | |
| 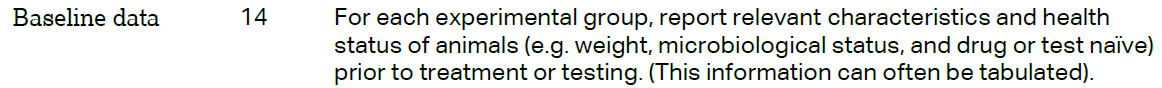 | Methods Chapter 3, Paragraph 1 | |
| 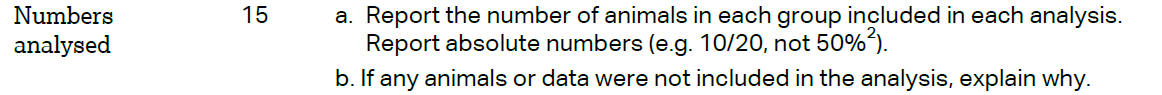 | Methods Chapter 3, Paragraphs 2, 4 | |
| 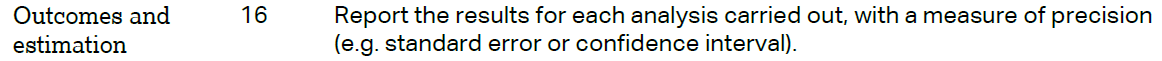 | Figure 3-4 | |
| 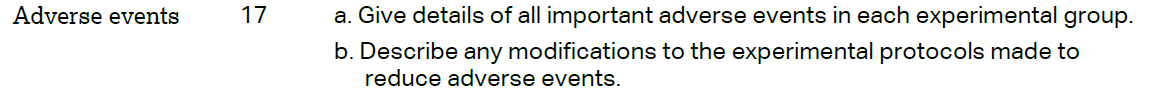 | Methods Chapter 3, Paragraph 2 | |
| DISCUSSION |  | |
| 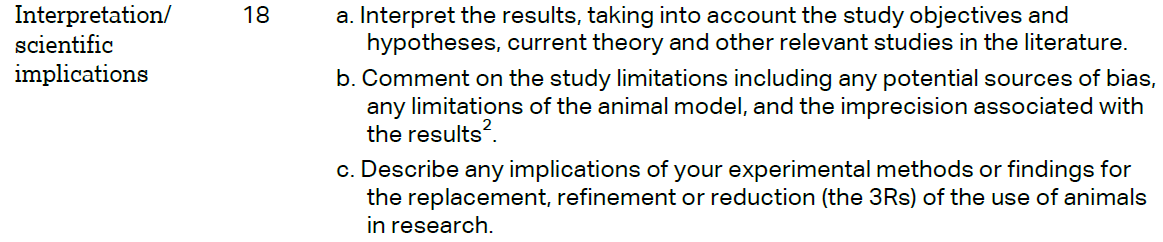 | Throughout Results and Discussion | |
| 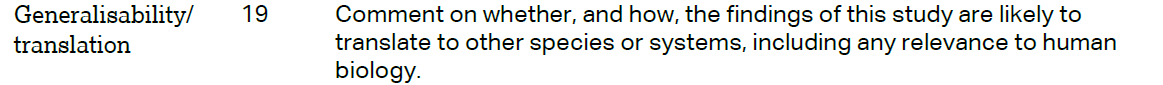 | Conclusion | |
| 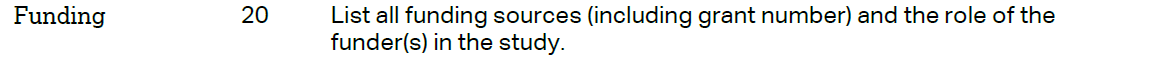 | | Funding |


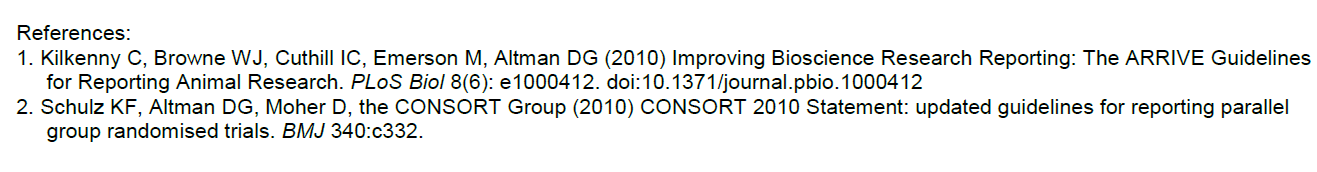

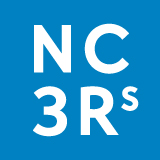

Supplement: S6 Fig — (DOCX) [file pone.0204776.s006.docx]
